# Ethnobotanical study of medicinal plants used in Artuma Fursi district, Amhara Regional State, Ethiopia

**DOI:** 10.1186/s41182-022-00438-z

**Published:** 2022-11-09

**Authors:** Mohammed Yimam, Siraj Mammo Yimer, Tamirat Bekele Beressa

**Affiliations:** 1 Department of Biology, College of Natural and Computational Science, Ambo University, P.O.Box 19, Ambo, Ethiopia; 2 Department of Pharmacy, College of Medicine and Health Sciences, Ambo University, P.O. Box 19, Ambo, Ethiopia

**Keywords:** Artuma Fursi, Ethnomedicinal, Indigenous knowledge, Herbal medicine, Human ailment

## Abstract

**Introduction:**

Indigenous people of different ethnic groups in Ethiopia are noticeably reliant on traditional medicinal plants for their healthcare due to their effective medicinal values. The study was aimed to document different herbal medicinal plants used and the associated knowledge of herbal medicine in the communities of the Artuma Fursi district.

**Methodology:**

Ethnobotanical data were collected through semi-structured interviews, field observations, focused group discussions with the informants selected from the study area. Key informants were selected by purposive sampling technique, while the rest, were selected by random sampling techniques. The collected data were analyzed using descriptive statistics; paired comparison, preference ranking, and informant consensus factor.

**Results:**

A total of 86 informants participated in the collection of the ethnobotanical data. A total of 92 medicinal plants were collected and identified. Fabaceae was the highest family cited (11.9%). The study revealed that leaves (31.1%), seeds (19.8%), and roots (12.26%) were the most cited plant parts used for the preparation of herbal medicine by the respondents. The most common method of preparation of herbal medicines was pounding (21.6%) and the most common route of administration was oral route (53.7%). The majority of the medications (60.3%) were prepared without the additive. Charcoal production was the major threat to medicinal plants in the study area.

**Conclusion:**

Artuma Fursi district is rich in medicinal plant and the associated indigenous knowledge. The documented knowledge will be helpful for further research in the drug development process.

## Introduction

Indigenous people living in different parts of the world have accumulated their local knowledge of plant resources and their uses as herbal medicines for many centuries. In Ethiopia, indigenous people of different ethnic groups are particularly dependent on traditional medicinal plants for their health care due to their effective medicinal value [1]. Traditional medicine refers to knowledge, skills, and practices based on theories, beliefs, and experiences indigenous to different cultures, used for the maintenance of health as well as in the prevention, diagnosis, improvement or treatment of physical and mental illness [2]. It plays a significant role in the fulfillment of primary health care needs in developing countries. Medicinal plants which are the basis for traditional medicine provide valuable contributions in treating humans and animals ailments [3]. Herbal medicines are used all over the world and depend on locally existing and available plant resources, which are simply accessible, simple to use, and affordable [4].

Medicinal plants are those that have active ingredients that help to relieve pain or heal ailments [5]. Due to the significant contributions of traditional practitioners, it has become well-known all across the world [6]. In developing countries, up to 80% of the population relies on medicinal plants for their primary healthcare needs [7]. Traditional medicinal plants are widely used in Ethiopia due to the inadequate coverage of the modern medical system, the scarcity of pharmaceuticals [8], the unaffordability of modern medicine [9], as well as the easy accessibility of traditional medicine.

Because of its long history, traditional medicine has become a vital part of the country's culture. Indigenous peoples in many parts of the country have created their own unique understanding of how to use, manage, and conserve plant resources [10]. It is well-known that traditional medicine knowledge is passed down orally from generation to generation, and that crucial information about the use of plants, such as the part used, mode of drug preparation, method of administration, diseases treated, and others, may be lost or discarded during this process [11].

Traditionally, plants were extraordinarily used in many societies, and are prevalent in African communities who lived in harmony with the natural resources for centuries without bringing any damaging effect on the survival of the biodiversity [12]. However, the survival and lifestyles of indigenous peoples and their long-term accumulated knowledge face challenges because of modernization, genetic erosion of plant and animal resources, low recognition of their knowledge and varied culture, and loss of biodiversity [13].

The current loss of medicinal plants in Ethiopia is due to natural and human-made factors, which are linked to the loss of vital indigenous knowledge of plants [14]. This has an impact on the long-term viability and continuation of traditional medicines, owing to the extinction of medicinal plant species [15]. On the other hand, the growth of contemporary education has exacerbated the loss of knowledge, causing younger generations to underestimate its traditional value. The people who attended modern schools are unwilling to learn from their parents, this is evidence of traditional wisdom steadily vanishing [16]. It is critical to document traditional medicinal plant applications to conserve traditional medicinal plant knowledge [17].

Communities in the Artuma Fursi district, like other communities in Ethiopia, are utilizing herbal medicines to treat both human and animal diseases, but there has not been any scientific research conducted to document the plant use knowledge of the local people to treat various human and livestock diseases. More ethnobotanical investigations are needed to document indigenous medical knowledge in the country [18]. Hence, the current study focused on documenting traditional medicinal knowledge and recording the list of medicinal plants used to treat human and animal diseases in the Artuma Fursi district.

## Materials and methods

### Description of the study area

**Fig. 1:**
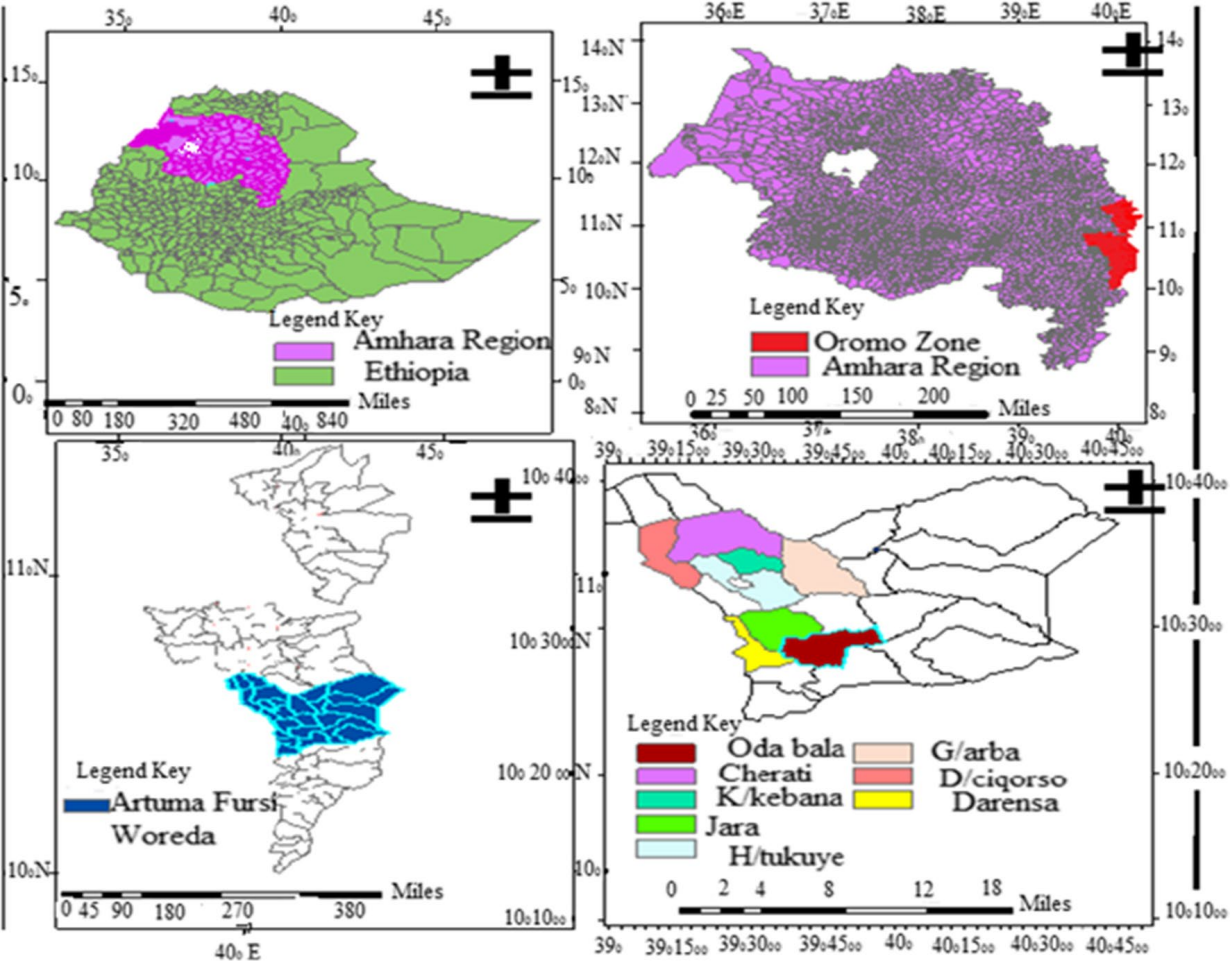
Location of the study area in Artuma Fursi district

Artuma Fursi is a district of the Oromo Nation Administrative Zones in Amhara Regional State, Ethiopia's (Fig. 1). It lies 302 km northeast of Ethiopia's capital, Addis Ababa, and 525 km southeast of Bahir Dar, the region's capital. Its absolute coordinates are 10°30′30″–10°34′0″N and 39°55′0″–39°58′30″E and it is bordered on the South Jile, on the West North Shewa Zone, on North Dewe Harawa, and from the East by Afar region, its capital is Chefa Robit town. The district has all four climatic zones (arid, semi-arid, semi-humid, and humid).The highest rain fall received in summer and followed by spring. The mean annual rain fall is 1035 mm [19]. Based on the 2007 national census of Ethiopia; the district had a total population of 82,842, of whom 40,938 are men. The majority of the populations were living in the rural area (92.8) and Muslim (97.76%) [20]. There are 6 health centers and 25 health posts in the district.

### Ethnobotanical data collection

The ethnobotanical data were collected from March 2020 to July 2020. The techniques employed in collecting ethnobotanical data included a semi-structured interview, field observation, and guided field walks with informants to obtain medicinal plants of the locality.

Key informants were selected by purposive sampling technique, while the rest, respondents were selected by random sampling techniques.

Information about the medicinal plant's local name, plant parts used methods of collecting and preparation, disease treated, the dosage used, route of administration, ingredients added, whether it is wild/cultivated were recorded during the study.

### Ethnobotanical data analysis

Ethnobotanical data gathered through semi-structured interviews and field observation, was analyzed using descriptive statistics; paired comparison, preference ranking, and informant consensus factor [10, 21, 22].

#### Informant consensus factor (ICF)

An ICF was performed to establish the relative importance of each use directly from the degree of agreement among respondents. The disease categories were identified based on local explanations, causes of disease and symptoms treated, and the informant consensus factor was calculated for each disease category. The ICF was calculated as follows: ICF = (nur − ns)/(nur − 1), where, ICF = informants consensus factor nur = number of use citation in each category, ns = number of species used [23]. The factor provides a range of 0 to1, where a high value acts as a good indicator for high rate of informant consensus.

### Preference ranking

A preference ranking was conducted following G Martin [21]. When a variety of plant species are utilized to treat the same health problem, individuals prefer one over the other. Key informants were given the task of comparing the given medicinal plants based on their value, with the highest number (5) given to the medicinal plants they preferred to be the most effective in treating the selected disease and the lowest number (1) given to the medicinal plants they preferred to be the least effective in treating the selected disease.

### Paired comparison of medicinal plants

A paired comparison was made for five medicinal plants used to treat stomach aches in the study area. Ten key informants were allowed to give rank to these medicinal plant species based on their efficiency as follows: 1 = least, 2 = good, 3 = very good and 4 = excellent.

### Direct matrix ranking

Direct matrix ranking was conducted following G Martin [21] and CM Cotton [10] in order to compare the versatile use of a given plant species based on the information gathered from informants. The multipurpose use of plant species includes such as use for food, medicine, firewood and charcoal. Six key informants were selected and ordered to assign use values to each of the attributes. Each chosen key informants was asked to assign use values (5 = best, 4 = very good, 3 = good, 2 = less used, 1 = least used, and 0 = not fencing and furniture). The average score was summed up and ranked.

## Results

### Information about respondents in the study area

Information was collected from 86 respondents (70 males and 16 females) using a semi-structured interview, field observation, and guided field walks. The respondents were sorted into three age groups: young (20–34), 16(18.6%); middle-aged (35–49), 28(32.55%); and elders (50–80), 42(48.83%). Out of this, the elders were dominant.

### Medicinal plants in the Artuma Fursi district

A total of 92 traditional medicinal plant species were collected and identified for treating human and animal disorders. They were divided into 87 genera and 45 families. The Fabaceae family has contributed the most medicinal plant diversity (11.95%), followed by the Euphorbiaceae family 6 (6.52%) and two families Solanaceae and Rutaceae each represented 5(5.4%). The remaining families are placed according to the species they contain.

### Medicinal Plants used for the therapeutics of different ailments

Among 92 plant species recognized from the study area, 74 (80.4%) species were used to cure human disease (Table 1), whereas, 10 (10.8%) species were used for animal ailments (Table 3) and 8 (8.69%) species were used for both human and animal (Table 4).

**Table 1:** List of medicinal plants used to treat human ailment by Artuma Fursi district

Scientific name	Family	Local name	Plant parts used	Disease treated by plant	Route of administration	The way the plant used	How to prepare the medicines
*Acacia etbaica* Schweinf	Fabaceae	Girar	R	Evil eye	Nasal	Dry/fresh	Water is added to the root after crashed. After then, a few drops of juice are inhaled
			SB	Wound	Topical	Dry	The stem bark is roasted, powdered, and sprayed over the wound
			R	Diarrhea	Oral	Dry	One cup of powdered dried root with water is taken
*Allium cepa* L	Liliaceae	Keyshinkurt	Bb	Hypertension	Oral	Fresh	The bulb is cut and macerated in water, filtered before being consumed
*Allium sativum* L	Alliaceae	Nechshinkurt	Bb	Common cold	Inhalation	Fresh	The bulb is powdered and smelled
				Malaria	Oral	Fresh	The bulb is crushed, combined with butter and pepper powder
*Aloe macrocarpa* Tod	Aloaceae	Wonde Eret	L	Impotency	Oral	Fresh	The leaf is cut in to pieces and taken with leaf
				Emaciation	Topical		The latex is combined with butter and placed on the penis, after which it is heated by the fire for many days
*Artemisia abyssinica* Sch.Bip. exA.Rich	Asteraceae	Ariti	L	Stomach ache	oral	Dry	The juice of the crushed leaves is combined with water or honey and taken orally
*Brassica carinata* A.Braun	Brassicaceae	Gomenzer	SD	Cancer	Topical	Dry	The seed is pounded and mixed with honey
*Arachis hypogaea* L	Fabaceae	Lewuz	SD	Cough	Oral	Dry	The dry seed is mashed and cooked in water
*Brassica nigra* (L.) Koch in Rohling	Brassicaceae	Senafich	SD	Stomach ache	Oral	Dry	The decoction of the dried seeds and *Lepidium sativum* seeds prepared and taken
*Calpurnia aurea* (Aiton) Benth	Fabaceae	Digita	L	Body lice	Topical	Fresh	The leaf is pounded and soaked in water, is used for bathing
				Malaria	Oral		The leaf is crushed and mixed with garlic leaf and rue fruit and soaked in water
*Capparis tomentosa* Lam	Capparidaceae	Gimero	SB	Epidemic	Inhalation	Fresh	The bark is pounded and fumigated
			L	Asthma	Oral	Fresh	Decoction is made from the leaves
*Capsicum annuum* L	Solanaceae	Mitmita	Fr	Amoeba	Oral	Fresh/dry	The fresh fruit or dry is added to food, meat and eaten
*Carica papaya* L	Caricaceae	Papaya	Fr	Malaria	Oral	Fresh	The fruit is crushed and mixed with water
*Carissa spinarum* L	**Calotropis procera (Ait.) Ait.f. [family** Apocynaceae	Agam	R	Evil eye	Inhalation	Dry	The dried root fumigated
			SD	Eye infection	Topical		Mixed with the charcoal powder, fresh butter and water, and then applied to the affected part of the eye
*Calotropis procera* (Ait.) Ait.f	Asclepiadaceae	Yeginkuas	Lx	Hemorrhoid	Topical	Fresh	The latex is applied to the anus
*Carum copticum* D.C	Umbelliferae	Nechazmud	SD	Stomach Discomfort	Oral	Dry	Mixed with red pepper (to reduce hotness)
*Cicer arietinum* L	Fabaceae	Shimbra	Wh	Malaria	Oral	Dry	The whole plant crushed, boiled and drunk
				Leech	Oral	Fresh	Smashed, mixed in water and given for cattle
*Citrus limon* (L.) Burm.F	Rutaceae	Lomi	F	Hypertension	Oral	Fresh	Fruit juice is mixed with tomato
*Citrus aurantium* L	Rutaceae	Komtate	F	Amoeba	Oral	Fresh	The fruit is consumed in the morning for 10 days
*Citrus medica L*	Rutaceae	Terengo	L	Pain attack	Oral	Fresh	The leaves boiled and filtrate is drunk
*Citrus aurantiaca* Swingle	Rutaceae	Birtukan	L	Measles	Topical	Fresh	The leaf is mixed with seed of *Guizotia abyssinica***.** Then the mixture is applied on the affected part of the body
***Clerodendrum myricoides*** **(**(Hochst.) R. Br. ex Vatke	Lamiaceae	Misrich	F&L	Malaria	Oral	Dry	The leaf and fruits, bulb of garlic, fruits and leaf of rue are mixed powdered and soaked in honey for one day and one glass per day is taken
			L	Vomiting		Fresh	Five leaves is crushed, pressed, and drank after being beaten with water
			R	Constipation			Crushed and pounded and then given orally
				Evil eye	Oral	Fresh	Squeeze and drunk orally
*Clematis hirsute* Guill. &Perr	Ranunculaceae	Azohareg	L	Leishmaniasis	Topical	Fresh	The leaf is pounded, and applied on the affected area with salt
			R	Hemorrhoid			After pounding and roasting; it is applied on the affected area
*Coffea arabica* L	Rubiaceae	Buna	F	wound	Topical	Dry	The roasted powder is applied to the wound
			SD	diarrhea	Oral	Dry	The powder is mixed with honey and eaten
*Combretum collinum* Fresen	*Combretaceae*	Woyba	SB	lower back pain	Topical	Fresh	Powdered fresh stem bark is applied on the place of pain
				Cosmetics			The fresh stem bark put in fire; fumigated to the whole body part
*Combretum molle* R.Br.exG.Don	Combteraceae	Abalo	SD	Measles (Chiffe)	Topical	Dry	Its seed is crushed powdered and mixed with butter and applied on the affected part until recovery
*Cordia africana* Lam	Boraginaceae	Wanza	SB	Jaundices	Oral	Dry	The stem bark is pounded before being boiled in milk. One glass is given orally
			S	Urinary incontinence			Pounded dry seed is combined with water. One cup is given per day
			Lx	Gastritis		Fresh	The latex is given on an empty stomach
			SB	Leg Wound	Topical	Fresh	The stem bark is heated and placed to the wound
*Croton macrostachyus* Del	Euphorbiaceae	Bisana	L	Ring worm	Oral Topical	Fresh	Prepare the juice in water and apply it to the affected area of the body
			L	Wound		Dry	The shot powder is combined with butter and applied
			SB	Malaria	Oral	Dry	One glass of powdered skin bark mixed with honey is consumed orally
				To stop bleeding	Topical	Dry	Squeeze and tie on the area
			LX	Wound	Topical	Fresh	Applied to the wound
*Cucumis ficilolious* A.Rich	Cucurbitaceae	Yemidirembuay	R	Diarrhea	Oral	Fresh	The root is crushed and mixed with water before being allowed to drink
*Cucurbita pepo* L	Cucurbitaceae	Duba	SD	Tape worm	Oral	Dry	It is used to treat tape worm in women who are pregnant. The seeds will be eaten
*Dichrostachys cinerea*(L.)	Fabaceae	Ader	SB	Scorpion bite	Topical	Fresh	The crushed fresh stem bark is applied to the afflicted area
*Datura stramonium* L	Solanaceae	Astefaris	L	Baldness	Topical	Fresh	After pounding and squeezing the leaves, it is applied to the Scalp
			S	Tooth ache	Oral	Fresh/dry	The seed is ignited, and the resulting smoke is inhaled
Dodonaea angustifolia L.f	Sapindaceae	Kitkita	L	Bone Fracture	Topical	Dry	The leaf is crushed, powdered, combined with butter, and applied to the wound as a cream
				Wound			The leaf is chopped, powdered, and combined with butter, administered to the affected area
				Dysentery		Fresh	The leaf is soaked in water then filtered. The filtrate is given orally with sugar
*Dovyalis abyssinica* (A.Rich.) Warb	Flacourtiaceae	Koshim	S	Joint pain	Topical	Fresh	The seed is pounded and combined with *Citrus aurantifolia* latex. The mixture is then tied around the injured leg
*Echinops kebericho* M	Asteraceae	kebercho	R	Evil eye	Inhalation	Dry	The dried root is crushed; put in fire and the smoke is inhaled
*Ehretia cymosa* Thonn	Boraginaceae	Wulaga	L	Leech	Nasal	Fresh	The fresh leaf is pounded, squeezed then applied nasally
				Toothache	Oral	Fresh	The leaves of *Psidium guajava* and *Calpurnia aurea* are crushed and mixed with the leaves of *Ehretia cymosa*
*Euclea racemose* (DC) Dandy	Ebenaceae	Dedeho	SB	Tooth ache	Oral	fresh	For a while, biting the stem bark between my teeth
			L	Tape worm			Crushed and combined with water, decanted before being consumed
*Euphorbia tirucali* L	Euphorbiaceae	Kinchib	LX	Hemorrhoid	Topical	Fresh	The latex is applied on the affected part
*Euphorbia platyphyllos* L	Euphorbiaceae	Anterfa	L	Leshmaniasis	Topical	Fresh	The latex is applied on the affected part
*Lantana camara* L	Verbenaceae	Eregnakolo	L	To stop bleeding	Topical	Fresh	The leaf is pounded and tied around the area of the body that is bleeding
*Ficus sur* Forssk	Moraceae	Sholla	F	Itching	Topical	Fresh	A mixture of ripe fruit juice and butter is applied on the body
			F	Heart disease	Oral		The Boiled fruit eaten continuously
*Ficus vasta* Forssk	Moraceae	Warka	SB	Eczema	Topical	Fresh	The infusion of the bark is administered to the afflicted area
*Guizotia abyssinica* (L.F)Cassi	Asteraceae	Nug	SD	Cough	Oral	Dry	The dried seed is pounded, and mixed with sugar or honey and drunk
*Hordeum vulgare* L	Poaceae	Gebis	SD	Diarrhea	Oral	Dry	Seeds are immersed in water and allowed to germinate before being dried, roasted, and pulverized. The powder is then heated in water and drunk till the pain subsides
*Helianthus annus* L	Compositae	Suf	SD	Cough	Oral	Dry	The seed is pounded and boiled. Then drunk orally
*Impatiens tinctoria* Hook.f	Balsaminaceae	Ensosila	Rh	Rheumatism	Topical	Fresh	Crushed and roasted rhizomes are administered to the affected area
*Jasminum grandiflorum* L	Oliaceae	Tembelel	L	Tape worm	Oral	Dry	One spoon full fine powder is mixed with water and then drunk per day until you get relieve
*Kalanche petitiana* A.Rich	Crassulaceae	Endahula	L	Bugunji	Topical	Fresh	The leaf is pressed with water and put on the swollen skin
			R	Tonsillitis	Oral		The root is crushed and pressed with water and a cup is drunk
				Wound	Topical	Fresh	Placed on fire and tied on affected site
				Ascariasis	Oral	Fresh	Squeezed and drunk half cup
*Lagenaria siceraria* (Mollina) Standl	Cucurbitaceae	Qil	L	Ear infection	Topical	Fresh	The ear is irrigated with the water from the leaf
*Lawsonia inermis* L	Lythraceae	Hina	L	Dandruff	Topical	Dry	The leaf is mashed and combined with water then applied
*Lens culinaris* Medik	Fabaceae	Misir	SD	Cough	Oral	Dry	Dried seed and leaf of decoction is given orally
*Lepidium sativum* L	Brassicaceae	Feto	SD	Dysentery	Oral	Dry	The seed is pounded, mixed with yoghurt; shaken well and drunk
*Linumu sitatissimum* L	Lineaceae	Telba	SD	Wound	Topical	Dry	The seed is mashed, mixed with honey, and applied to the wound as a bandage
*Lippia adoensis* Hochst. ExWalp	Verbenaceae	Kesse	L	Eczema, fungal infection	Oral	Fresh	fresh leaf juice mixed with a small amount of water
				Common cold	Oral	Fresh	Fresh leaf is pound, diluted with water and given orally
*Lycopersicon esculentum* Mill	Solanaceae	Timatim	Fr	Heart disease	Oral	Fresh	The fresh tomato is eaten occasionally in the morning
*Melia azedarach* L	Meliaceae	Mim	L	Abortion	Oral	Fresh	Squeezed and drunk
				Malaria	Oral	Fresh	Squeezed and drunk a cup
*Musa X paradisiac* L	Musaceae	Muz	Fr	Headache	Topical	Fresh	The fruit's skin is pilled and tied around the skull
*Moringa oleifera* Lam	Moringaceae	Shiferaw	L	hypertension	Oral	Dermal	Crushed, powdered leaves are combined with heated water
*Myrtus communis* L	Myrtaceae	Ades	L	Stomach ache	Oral	Fresh	Chewing and taking the sap
				Scabies	Topical	Dry	A dry powder is combined with butter and applied on affected area
*Nigella sativa* L	Ranunculaceae	Tikur-azmud	SD	Headache	Inhalation	Dry	The seeds are wrapped in a clean piece of cloth and sniffed after being combined with melted butter
*Ocimum basilicum* L	Lamiaceae	Zikakibei	L	Headache	Oral	Fresh	Fresh leaf juice is given orally
				Malaria			Fresh juice is given orally
				Stomach ache			Fresh leaf is given for chewing and swallowed
*Ocimum lamiifolium* Hochst.exBenth	Lamiaceae	Demakesse	L	Sunstrike	Oral	Fresh	One cup of the leaf is given orally after it has been pressed with water
				Head ache	Inhalation		The leaf is boiled and inhaled
				Fibril illness	Topical	Fresh	The leaf is squeezed, bathed with it
				Common cold	Oral	Fresh	Squeezed and drunk
*Olea europaea* subspp cupsidata L	Oleaceae	Woyra	L	Eye irritation	Topical	Fresh	The leaf is crushed and pressed with water and applied to the eye
			L	Headache	Topical	Fresh	The oil mixed with powdered *Echinops kebericho* and placed on head
			Lx	Asthma	Oral	Fresh	The oil mixed with honey and 1/2 coffee cup is taken
*Pisum sativum* L	Fabcceae	Ater	SD	Bugunji(Boils)	Dermal	Dry	The seed is pounded and placed on the wound until disappearance of the swelling
*Psidium guajava* L	Myrtaceae	Zeytune	F	Dysentery	Oral	Fresh	Amoebic dysentery can be treated using the fruit
*Podcarpus falcatus* (Thunb.) Endl	Podocarpaceae	Zigba	L	Epilepsy	Inhalation	fresh	The dry part of the leaf is fumigated after it has been crushed into smaller bits
*Rhamnus prinoides* L’Herit	Rhamnaceae	Gesho	L	Scabies	Topical	Dry	The damaged skin is treated with a mixture of powdered leaves and butter
				Tonsillitis	Oral	Fresh	The fluid is swallowed once the leaf is chewed
*Rumex nervosus* Vahl	Polygonaceae	Embacho	L	Eye disease	Topical	Fresh	The aye lash is sprayed with the juice collected from the leaf
				Circumcision	Dry		The leaf extract is combined with warmed butter and applied to the wound
*Ruta chalepensis* L	Rutaceae	Tenadam	ST	Common cold	Oral	Fresh	The leafy branch is steeped in coffee and consumed
				Malaria			The branch is boiled with zinger and garlic and then consumed on a daily basis
*Saccharum officinarum* L	Poaceae	Shenkoraageda	ST	Gastritis	Oral	Fresh	Chewing in the mouth the swallowing the juices
*Schinus molle* L	Anacardiaceae	Kundoberberie	Fr	Jaundices	Oral	Dry	The fruit crushed and soaked with milk. One glass is taken daily with *Solanum nigram* fruit
*Senna didymobotrya (Fresen.) H.S.Irwin&Barneby*	Fabaceae	Yeferenjdgta	SD	Diarrhea	Oral	Dry/fresh	The seed is crushed and roasted, then drunk with coffee
*Sida schimperiana* Hochst.exA.Rich	Malvaceae	Chifrig	L	Eye defect	Topical	Fresh	After combining the leaf with water and squeezing it, the juice is administered as a drop till the patient recovers
*Sesamum indicum* L	Pedaliaceae	Selit	SD	Deafness	Topical	Dry	The seed is combined with *Guizotia abyssinica* seed, pounded, and a small amount of water is added
*Solanum incanum* L	Solanaceae	Enbuay	R	Swelling	Topical	Fresh	The root is mashed, then combined with honey and wrapped in fabric around the diseased area of the body
				Abdominal pain	Oral		The fluid is swallowed after chewing the root
				Tonsillitis	Oral	Fresh	The seed are squeezed and taken orally
				Stomach ache	Oral	Fresh	The root is chewed and swallowed
*Tragia cinerea* (Pax) Radel	Euphorbiaceae	Aleblabit	R	Evil eye	Inhalation	Dry/ fresh	Inhale smoke from a dried or fresh root that has been placed on a fire
*Trigonella foenum-graecum* L	Fabaceae	Abish	SD	Varicose vein	Oral	Dry	The seed is pulverized, combined with honey, and thoroughly shaken. It is eaten regularly
*Vicia faba* L	Fabaceae	Bakela	SD	Swell	Topical	Fresh	The seed is spat on the affected area after being crushed by the teeth
*Ximenia caffra* Sond	Olaaceae	Enkoy	SB	Herpeszoster‟	Topical	Dry	Butter is used to apply the powdered bark to the affected area
*Zingiber officinale* Roscoe	Zingiberaceae	Zinjible	Rh	Stomach ache	Oral	Dry/fresh	The bark is peeled off, diced, chewed, and the liquid ingested
*Zizipus spina-christi* L. Desf	(Rhamnaceae)	Kurkura	L	Dandruff	Topical	Fresh	The leaf is chopped and the scalp is washed by mixing with water

*R* root, *L* leaf, *SB *stem bark, *Fr* fruit, *SD* seed, *Sh* shoot, *St* stem, *Wh* whole plant, *Br* branches, *Rh* rhizome, *Bu* bulb, *Lx* latex

### Ethnomedicinal plants used to treat human disease

A total of 82 medicinal plant species belonging to 80 genera and 37 families were collected and documented which are frequently used for treating only human ailments in Artuma Fursi District (Table 1). Among the above families, Fabaceae is the leading and contains the highest number of species 11 (13.4%), followed by Euphorbiaceae 6 (7.3%), Rutaceae 4 (4.87%), Solanaceae 4 (4.87%), Asteraceae 4 (4.87%). Three families including, Cucurbitaceae, Brassicaceae, and Oleaceae contain 3 species each.

### Plant habitats and parts used to treat human ailments

**Fig. 2:**
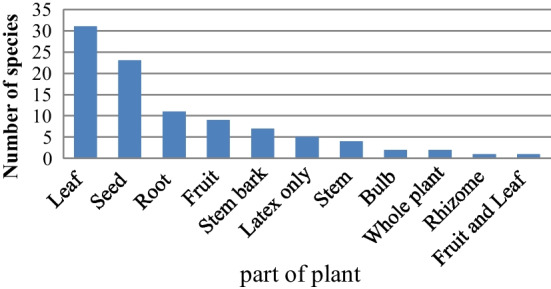
Plant part used for preparation of human remedy

Of all medicinal plants collected and identified, 43 plant species (52.4%), were from natural habitat, while 35 species (42.6%) were form home garden while 4 species (4.8%) were both natural habitat and home garden. The most commonly used plant parts were leaves 31 (32.29%), followed by seed 23 species (23.9%) and roots 11 species (11.4%) whereas 1 species (1.04%) was fruit and leaf (Fig. 2).The highest 41 species (50%) of the remedy were prepared from fresh plants while the remaining were in a dried 33 species (40.2%) and 8 species (9.7%) fresh or dried 8 species (9.7%).

### Methods of remedy preparation in the study area

**Fig. 3:**
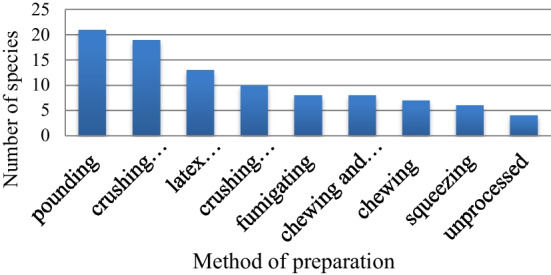
Preparation methods for medicinal plants used to treat human ailment

The highest method of medicinal plant preparation used to treat human disease was by pounding 21 species (21.9%) followed by crushing and squeezing 19 species (19.79%) and other forms of preparation are also indicated (Fig. 3).

### Dosage and route of administration

The most-reported route of applications was oral, 65 species (53.12%) followed by topical, 34 species (27.2%), inhalation 9 (7.2%), nasal, 3 species (2.4%).

### Additives or solvents

With regard to additives the majority of remedies, 54 species (56.25%) were prepared with no additives (Table 2).

**Table 2:** Additives or solvents used for human remedial preparations in Artuma Fursi district

Additives	Number of species	Percentage (%)
No additive	54	56.25
Water	23	23.9
Coffee	9	9.37
Butter	7	7.29
Honey	3	3.12

### Adverse side effects

The majority of the medicinal plant species were reported not to possess significant side effects at the administered doses, in which 78 species (81.25%) species with no adverse side effects were followed by 14 species (14.5%) pain, 2 species (2.08%) fever, 1 species (1.04%) frequent urine and 1 species (1.04%) diarrhea.

### Ethnoveterinary medicinal plants in Artuma Fursi District

A total of 10 Ethnoveterinary medicinal plants species used to treat only animal disease (Table 3). These species belonging to 7 genera and 7 families were recorded in Artuma Fursi District. Family Euphorbiaceae was dominant contained 2(20%) followed by Agavaceae, Polygonaceae, Solanaceae, Cucurbitaceae, Acanteraceae and Moraceae each represented by single species 1(10).Unlike that of human medicine traditional medicinal healers do not give equal weight for Ethnoveterinary remedy.

**Table 3:** List of ethnoveterinary plants used to treat animal ailments in the study area

Scientific name (family name)	Family	Local name	Part	Disease treated	Root of administration	The way the plant used	Way of preparation
*Arundo donax*(Hudson) Link	Poaceae	Shembeko	St	Bone fracture	Topical	Dry/fresh	Dried or fresh stem is applied through the affected organ and tilled
*Rumex nepalensis* Spreng	Polygonaceae	Tult	R	Loss of weight	Oral	Fresh	Crushed and then given to skinned cattle
*Ricinus communis* L	Euphorbiaceae	Gulo	R	Sudden sickness	Oral	Fresh	The root pounded and mixed with cold water
*Nicotiana tabacum* L	Solanaceae	Tinbaho	L	Leech	Oral	Fresh	The pounded leaf mixed with water, then given to drink
*Agave sisalana* Perrine ex Engel	Agavaceae	Kacha	LX	Leech	Nasal	Fresh	Its latex *is* mixed with the pounded leaf of *Plectranthus amboinicus,* then the fluid is filtered and given to the cattle through nose
*Maytenus arbutifolia* (A.Rich.) Wilezek	Celastraceae	Atat	L	Parasites	Oral	Fresh	Leaf decoctions are used to treat external parasites in both domestic and wild animals
*Iusticia schimperiana* Hochst. ex.Nees	Acanthaceae	Sensel	Wh	Laxative	Oral	Dry	The entire plant is crushed, pounded, and then combined with water before being consumed
*Gossypium borbadense* L	Malvaceae	Tit	L	Diarrhea	Oral	Fresh	Powdered and mixed with water and given to drunk
*Euphorbia abyssinica* Gmel Euphorbiaceae	*Euphorbiaceae*	Kulkual	LX	Rinderpest	Inhalation	Dry	Fumigating the affected cattle
*Ficus carica* L	Moraceae	Beles	L	Tail sore	Topical	Fresh	Latex of the plant applied on the tail sore /wound/ formed after operation
*Ficus vasta* Forssk	*Moraceae*	Warka		Loss of weight	Oral	Fresh	The leaf is crushed and boiled before being fed to skinned cattle

*R* root, *L* leaf, *SB* stem bark, *Fr* fruit, *SD* seed, *Sh* shoot, *St* stem, *Wh* whole plant, *Br* branches, *Rh* rhizome, *Bu* bulb, *Lx* latex

Growth form of most Ethnoveterinary medicinal plants were shrubs 6(60%) followed by herbs 3(30%) and least number of growth form used for the preparation of ethnoveterinary medicines are climbers 1(10%) in the district.

### Medicinal plants used to Treat both Human and Livestock Aliments

8 (8.69%) of species were used for both human and animal. The species used to treat both human and livestock aliments are *Capparis tomentosa* Lame., *Carissa spinarum L., Cicer arietinum* L., *Clerodendrum myricoides* (Hochst.) Vatke. *Croton macrostachyus* Del. etc (Table 4). Table 4 shows the name of species, parts used, diseases treated, route of administration, application and dosage.

**Table 4:** List of traditional medicinal plants used to treat both human and animal ailment

Scientific name	Family	Local name	Habit	Collection code	Source	Part	Used for	Diseas treated	RA	Cp	Way of preparation
*Capparis tomentosa* Lame.	Capparidaceae	Gimero	S	MY 10	W	SB	Ca	Epidemic	O/N	F	**The bark is crushed and placed on the red hot charcoal and used to fumigate smokes**
					L	R	HU	Asthma	O		*Decoction of the leaves is used for the treatment of asthma.*
*Carissa spinarum *L.	*Apocynaceae*	Agam	S	MY 13	W	R	HU	Evil eye	N	D	Fugmenting smoke of dried root
						SD	Ca	Eye infection	E		**The charcoal powder is mixed with fresh butter and water, and then stained the affected part of the eye.**
*Cicer arietinum*L.	*Fabaceae*	Shimbra	H	MY 17	HO	Wh	HU	Malaria	O	D	Powderd boiled and drunk
					SD		Ca	Leech	O	F	**Smashed, mixed in water and given for cattle**
*Clerodendrum myricoides* (Hochst.) Vatke.	*Lamiaceae*	Misrich	S	MY 22	HO & W	F & L	HU	Malaria	O	D	**The leaf and fruits, bulb of garlic, fruits and leaf of rue are mixed powdered and soaked in honey for one day one glass**
						L	HU	Vomiting		F	**Five leaves pound with water and crushed, squeezed drunk**
						R	Ca	Consepstion			**Crushed and pounded and then given orall**
						R	HU	Evil eye	O	F	**Squeeze and drink orally**
						SB	HU	Wound leg	O	F	**Heat on fire and put on the wound**
*Croton macrostachyus *Del.	*Euphorbiaceae*	Bisana	T	MY 28	W	L	HU	Ring worm	DM	F	**The shoot is crushed and squeezed in water then directly dropping the juices on injured part.**
						L	HU	Cut		D	**The shoot is crushed, powdered and mixed with butter and creamed injured parts**
						SB	HU	Malaria	O	D	**The steam bark is crushed, powdered soaked in honey and one glass is taken orally**
							HU	Blood clot	DM	D	**Squeeze and tie on the area**
						LX	Ca	Wound	DM	F	**Paint the wound area**
*Dodonaea angustiflia* L.f.	*Sapindaceae*	Kitkita	S	MY 33	W	L	CA	Bone fracture	D	D	**The leaf is crushed, powdered, mixed with butter and creamed the wound or affected part**
							HU	Wound			**The leaf is crushed, powdered, mixed with butter and creamed the wound**
							HU	Dysentry	O	F	**The leaf is crushed, soaked in water with sugar, decanted and one can is taken orally.**
							HU	Malria		D	**The leaf and fruits mixed with onefourth of bulb of garlic, fruits and leaf of rue powdered, soaked in honey and one glass daily**
*Ehretia cymosa* Thonn	*Boraginaceae*	5Wulaga7	T	MY 36	W	L	Ca	Leech	N	F	**The fresh leaves of** *Ehretia cymosa* **is pounded, squeezed then applied nasally**
							HU	Toothache	O	F	**Crushed and put with leaves of** *Calpurnia aurea*
*Ficus vasta* Forssk.	*Moraceae*	Warka	T	MY 42	W	L	Ca	Loss of weight	O	F	**The leaf is crushed, boiled and given for skinned cattle**
						SB	HU	Eczema	D		**The infusion of the bark is applied on the affected part**
						L	HU	Wound	DM	F	**Placed on fire and attach on affected site**
							HU	Ascaris	O	F	**Squeezed and drink half cup amount**
							HU	**Common cold**			**Fresh leaf pound is diluted with water and given orally**
							HU	**Common cold**	O	F	**Squeezed and drink in cup**
							HU	Malaria	O		**The branches are boiled with rhizome of zinger and bulbs of garlic in the tea and one cup is taken continuously**
							HU	Abdomenal pain	O		**The root is chewed and the fluid is swallowed**
							HU	Tonsillitis	O	F	**The seed are squeezed taken orally**
							HU	**Stomachache**	O	F	**Chew and swallow**

*S* shrub, *T* tree, *H* herb, *C* climber, *R* root, *L* leaf, *SB* stem bark,*Fr* fruit, *SD* seed, *Sh* shoot, *St* stem, *Wh* whole plant, *Br* branches, *Rh* rhizome, *Bu* bulb, *Lx* latex, *Uf* used for, *Hu* human, *Ca* cattle, *Ra* route of administration, *O* oral, *D* dermal, *N* nasal, *E* eye, *Er* ear, *An* anal, *Cp* condition of plant used, *F* fresh, *D* dry, *D/F* dry or fresh

### Informant consensus factor (ICF)

The study's findings revealed that diseases that are common in the study area have a higher level of informant consensus (IFC). A medicinal plant with a high ICF indicates the agreement among the informants in treating specific ailments and is well-known among community members (Table 5).

**Table 5:** ICF of the given diseases category

Type of diseases	Ns	Nur	ICF
Parasite, worm and gastro-intestinal disease	22	143	0.85
Dermatological problems	19	104	0.82
Swelling, hemorrhoids	17	37	0.55
Respiratory diseases	12	43	0.73
Insect bite and physical damages	7	18	0.64
Internal disease diabetes, hypertension and headache	16	50	0.69
Livestock diseases	19	57	0.67
Organ diseases ear, eye, heart	8	27	0.73
Genitourinary problems—gonorrhea and impotency, urine flow at night	5	7	0.33
Evil eye and sun strike	6	22	0.76
Problem of joint and bone	5	9	0.55

*Ns* number of species, *Nur* number of use report

### Preference ranking

The preference ranking was conducted for medicinal plants used for treatment of malaria. According to the respondents rank, *Allium sativum* was ranked first and *Clerodendrum myricoides* was ranked second (Table 6).

**Table 6:** Preference ranking of medicinal plants used for treating malaria

Name of species	Respondents (R1–R7)								
	R1	R2	R3	R4	R5	R6	R7	Score	Rank
*Calpurnia aurea*	3	2	3	4	2	4	2	20	4th
*Carica papaya*	5	3	3	2	4	2	3	22	5th
*Croton macrostachyus*	4	4	3	3	4	3	3	24	3rd
*Allium sativum*	4	4	5	4	4	4	5	30	1st
*Ocimum basilicum*	2	2	3	3	3	3	2	18	6th
*Clerodendrum myricoides*	5	3	4	3	4	3	2	25	2nd

### Direct matrix ranking for multiple uses of medicinal plants

Standard score for direct matrix ranking of six medicinal plants using a range of values from 0 to 5. 5 equals excellent, 4 equals very good, 3 equals good, 2 equals less, 1 equals least, and 0 equals not advantageous (Table 7).

**Table 7:** Standard score for direct matrix ranking of medicinal plants with use diversity

Major uses	Medicinal plants					
	*Acacia abyssinica*	*Cordia africana*	*Ficus sur*	*Croton macrostachyus*	*Olea europaea*	*Schinus molle*
Firewood	5	4	4	3	5	5
Medicine	3	3	3	4	3	3
Furniture	2	5	3	3	4	4
Construction	3	5	3	4	4	2
Charcoal	4	3	3	3	3	3
Forage	3	4	3	1	3	2
Edible fruit	0	3	2	0	0	0
Total	20	27	21	18	22	19
Rank	4th	1st	3rd	6th	2nd	5th

### Paired comparison on medicinal plants

The paired comparison test was conducted for medicinal plants used for treatment of stomach ache. *Ocimum basilicum* was chosen first, followed by *Zingiber officinale*, *Brassica nigra*, *Artemisia abyssinica*, and *Myrtus communis* (Table 8).

**Table 8:** Paired comparison on medicinal plants

Medicinal plants	Respondents										Score	Grade
	R1	R2	R3	R4	R5	R6	R7	R8	R9	R10		
*Artemisia abyssinica*	3	2	2	4	4	3	2	1	3	2	26	4th
*Zingiber officinale*	3	3	4	22	2	2	3	4	1	4	28	2nd
*Ocimum basilicum*	4	2	4	3	3	3	4	3	2	2	30	1st
*Myrtus communis*	3	2	3	2	1	3	2	4	1	1	22	5th
*Brassica nigra*	2	2	2	3	4	3	2	4	3	2	27	3rd

### Threats to medicinal plants in the study area

Man-made factors that influenced the medicinal plants in the area were charcoal, farming expansion, using trees for firewood, overgrazing, construction, and drought. The factors were ranked according to their degree of harm. Ten respondents were chosen to provide 5 of the most threatening factors and one of the least threatening. Therefore, charcoal was the most threatening factor, scoring 45, and the least threats to medicinal as supposed by informants were grazing, scoring 30 grading of main threats to TMP(R1–R10 = Respondents 1–10 and Values 1–5: 1 is the least destructive threat, and 5 is the most destructive one (Table 9).

**Table 9:** Threats to medicinal plants in the study area

Major threats	Respondents										Score	Rank
	R1	R2	R3	R4	R5	R6	R7	R8	R9	R10		
Grazing	3	2	4	3	4	2	4	4	2	2	30	6th
Construction	4	4	3	5	4	4	4	4	5	4	41	3rd
Agriculture	4	5	3	5	5	4	5	3	5	3	42	2nd
Charcoal	5	4	4	5	5	4	5	4	4	5	45	1th
Fire wood	3	4	5	3	3	5	5	3	4	3	38	4th
Drought	3	2	4	3	2	3	3	2	4	4	31	5th

## Discussion

Elders whose age ranged from 50 up to 80 years were knowledgeable respondents about medicinal plants because of many years of experience about plants than the other age classes, while young ones do not have the attention to understand the medicinal value of plants. In other similar studies conducted by [16, 17, 24] it was also reported that elders were the source of knowledge about medicinal plants. The majority of informants who participated in the interview do not read and write. This indicates that modern education has a greater impact on the loss of knowledge of medicinal plants. When someone gets a modern education they give less weight to traditional medicinal knowledge and they think about its side effects [25]. The majority of males (80.2%) are more knowledgeable than females (19.8%) which could be related to the country's traditional information transmission via the male line [26–28].

Fabaceae have contributed the highest medicinal plant diversity. This result is in line with that of [11, 29], who reported that Fabaceae is the leading family of plants that are used as medicinal plants. Fabaceae is one of the largest families which contributes medicinally important chemical components such as flavonoids, alkaloids, and coumarins [30]. Among the total of 81 species of ethnobotanical plants used to treat human disease and 11 species for animal disease herbs were prevalent, which accounts for 36 species (43.9%). The result was also similar with Megersa et al., kebede et al. and Tilahun et al. [11, 31, 32], who reported herbs as dominant growth form followed by shrubs and trees. However, this result is contrary to that ofG Alemayehu, Z Asfaw and E Kelbessa [33] who reported shrubs as the most used growth form in the preparation of remedies.

Most people, including herbalists in the study area, do not cultivate medicinal plants to keep their use confidential. In this regard, the finding was similar to that of A Kebede, S Ayalew, A Mesfin and G Mulualem [31] who conducted research in Dire Dawa city. A Tadesse, B Kagnew, F Kebede and M Kebede [34], also previously reported that most medicinal plants are mainly collected from wild habitats. The study was also greatly supported by the result of EL Molla [35] in which wild habitats were found to be a major source of traditional medicinal plants. In addition to this, scientific studies partly support the wild collection. The secondary metabolites are responsible for the medicinal value of plants, which need their natural environment under particular conditions of stress and competition that would not be expressed under cultivated conditions.

The plant parts most commonly used were leaves 31 (32.29%) and seed 23(23.9%). This research backs up the findings of Kebede et al. and Gebeyehu et al. [29, 31],who found that leaves were the most often used plant parts for making medicine treat human diseases. According to A Tadesse, B Kagnew, F Kebede and M Kebede [34]; M Giday and G Ameni [36] and F Mesfin, S Demissew and T Teklehaymanot [24], leaves were also the most commonly used plant parts followed by roots and seeds. The leaves are active in the process of metabolism and can be easily collected [37]. A highest 41 (50%) number of remedies were prepared from fresh plants and this finding agrees with the study conducted by Tadesse et al., Molla et al., Getaneh et al. [34, 35, 38], in which fresh preparation was greatly utilized for remedy preparation and these have active secondary metabolites significant for the treatment of disease rather than using dried forms of preparation.

The pounding was the highest method of medicinal plant preparation used to treat the human ailments. The pounding was a better way of preparation and no need for extra material to extract the active substances. The study was similar to the results of Tadesse et al.[34], who mentioned pounding as the major method of remedy preparation. The dosages of administration for human ailments in the area were different in terms of age, performance, and other criteria. The dosages were determined by using different local measurements such as cups, glasses, for liquid dosage forms, spoons for powder dosage forms, and fruits in number. A similar study conducted by Gebeyehu et al. and Molla et al. [29, 35] showed medicinal plants do not have an absolute dosage. The oral administration was the most popular and widely utilized mode of administration, followed by cutaneous (dermal) administration. A study conducted by Alemayehu et al. [33], in Minjar Shenkora district, also reported that the most commonly used route of administration was orally followed by dermal application. Oral route of administration is the simplest and continent route which could be used easily by traditional healer.

The majority of remedies 54 (56.25%) were prepared with no additives. This aligns with the study conducted by Mesfin et al. [39] in Gemad district. However, Getaneh et al. [38] documented the usage of additions such as butter and edible oil for wound and skin illness, as well as coffee, honey, and local beverages like Tela and Areke for plants with a bitter flavor.

The majority of human remedy preparations were harmless, in which 78 (81.25%) species with no adverse side effects. This study shows that most of the traditional medicines prepared by herbalists are free from adverse side effects, so that anyone can take the prepared medications without frustration [40]. But some other medicinal preparation have side effects like pain, frequent urine, fever, and diarrhea. For example, the leaf of *Clematis hirsuta* prescribed for leishmaniasis has serious pain E Hillenbrand [41].

The parasitic worm and gastrointestinal disease had a high ICF value (0.85), followed by dermatological (0.82). According to Heinrich et al. [23], high ICF values were crucial for identifying plants of special interest, in the investigation of bioactive chemicals. Some studies conducted in Ethiopia Hunde et al., Tamene et al., Abiyot et al. [16, 42, 43], have used the method of pair-wise ranking where informants make their choices on an individual basis. Preference ranking, paired comparison, and direct matrix ranking show the preference of medicinal plants over each other. This shows that those people obtain the knowledge via experience and differentiate medicinal plants that are successful in treating humans or their livestock diseases. Based on a preference ranking of six malaria-treating medicinal plants, the first rank was *Allium sativum*, which was the most effective medicinal plant for treating malaria. The study is in line with that of Abiyot et al. [43], in which *Allium sativum* was the most preferred anti-malarial plant.

Studies showed that shrubs were the most extensively utilized growth form in ethnoveterinary medicinal preparation followed by herb and trees. Similar findings showed in [44], show that higher utilization of shrubs followed by herbs in ethnoveterinary remedy preparation in Ankober District.

Some medicinal plants are versatile. It could be used for charcoal, food, firewood, construction, and furniture production. As shown in the study, *Cordia africana* and *Olea europaea* were ranked 1st and 2nd most chosen medicinal plants by the local community for a range of uses and are the most threatened species. The 3rd, 4th, 5thand 6th levels were for *Ficus sur*, *Acacia abyssinica*, *Schinus molle*, and *Croton macrostachyus*, respectively. This suggests that plants were overused for purposes other than medical formulations.

In the Artuma Fursi district, there is a loss of medicinal plants due to artificial factors such as deforestation for different purposes like charcoal. In the study area, many people are economically dependent on charcoal production to fulfill their needs and farming expansion due to population growth. Other main reasons for the loss of medicinal plants in the study area include firewood, construction, grazing, and drought. This study was contrary to the study done by [34], in Guduru district, who identified agricultural growth as the major danger to medicinal plants, followed by firewood and charcoal. The key subjects regarding threats to medicinal plants in the Amaro district were deforestation, followed by agricultural expansion, fire, charcoal trading, firewood collection, overgrazing, and drought [39].

## Conclusion

The study showed a variety of medicinal plants and traditional knowledge about how to use, prepare and administer by the local community of the Artuma Fursi district. The district has a rich diversity of medicinal plants for the management of human and livestock ailments many of which belong to the Fabaceae family. The plant species reported needs further study for the validation of the claimed pharmacological activities. Additionally, phytochemical screening which is guided by bioactive test is also needed to know the active compound in the reported medicinal plants. Medicinal plant species used in the district were collected from the wild which leads to the over exploitation without limitation. Therefore, awareness creation has to be implemented for the local communities and traditional herbalists on the sustainable use of plants and to cultivate medicinal plants around their homes.

## Data Availability

All data generated or analyzed during this study were included in this manuscript for publication.
